# *M. leprae, M. lepromatosis*, and Leprosy: Surprises and Surmises

**DOI:** 10.4269/ajtmh.26-0285

**Published:** 2026-06-03

**Authors:** David M. Scollard

**Affiliations:** National Hansen’s Disease Programs (USA), Baton Rouge, Louisiana, USA

In the clinic, the diagnosis of leprosy is often a surprise, as seen in the case report from Olson et al., reported in this issue of the *American Journal of Tropical Medicine and Hygiene*.^1^ They report the diagnosis of leprosy in a 75-year-old man who presented with a slowly growing erythematous plaque on his thigh, followed by nodular lesions that ulcerated. He lived in the Pacific northwest of the United States and Canada, and had no history of foreign travel or contact with any known case of leprosy. He had a long history of hunting but no contact with armadillos.

The laboratory was surprised, too, to find that Ziehl Neelsen (ZN) stains were negative and polymerase chain reaction (PCR) test for *M. leprae* was also negative. Further molecular testing revealed *M. lepromatosis* (a second leprosy pathogen discovered <20 years ago) was the causative agent. Both *M. leprae* and *M. lepromatosis* are weakly acid-fast and indistinguishable microscopically. Although molecular identification of *M. leprae* DNA began in the 1990’s using PCR to target various DNA sequences, *M. lepromatosis* was not identified until 2008, when tests of specimens from two patients with leprosy were negative for *M. leprae*.^2^ This experience has been repeated often; with increasing use of PCR as a diagnostic tool. *M. lepromatosis* is usually found only after testing for *M. leprae* is negative.

Olson’s patient presented with a localized lesion, highlighting the fact that *M. lepromatosis* can be found in all types of leprosy lesions, across the immunopathological spectrum from tuberculoid to lepromatous.^3^ It is not specifically associated with the highly-infected dermatological variant of lepromatous leprosy known as diffuse lepromatous leprosy, as was originally hypothesized.

Leprosy is rare in developed nations, and it is uncommon even in many parts of the world that are described as endemic. A prevalence near or below 1/10,000 people, the WHO target for “elimination of leprosy as a public health problem”, is a level sufficiently low that many physicians will never see a case beyond an experience in medical school. Even when low prevalence is achieved, surveillance must continue and such low prevalence poses major challenges for clinical awareness. The diagnosis may only be realized after several visits to multiple physicians. Unfortunately, for patients, leprosy is often a surprise to their doctors.

With respect to treatment and prognosis—whether leprosy is due to *M. lepromatosis* or *M. leprae—*may be a distinction without a difference, based on the evidence to date. The clinical and pathological manifestations of both infections are so similar that leprosy appears to be caused by two different but closely related species of mycobacteria.^4^ Both respond well to antimicrobial agents in the standard multiple drug treatment regimen used for leprosy: rifampin, dapsone, and clofazimine.

The difference between these two organisms may be more important from a public health perspective than for patient care. Both mycobacterial species have evolved to occupy the same unique niche: they are obligate intracellular organisms that prefer a relatively low growth temperature and can infect human peripheral nerves. Genomic studies indicate these species diverged from a common ancestor before the existence of *Homo sapiens*.^5^ *M. lepromatosis* existed in the Western hemisphere before humans were present, and long before *M. leprae* was introduced by humans through migration and slave trafficking.

Regardless of its “head start”, *M. lepromatosis* has remained an uncommon cause of human infection. The evidence base for comparing the frequency of human infection with these two pathogens only dates to 2008, but *M. lepromatosis* has been found in fewer than 5% of specimens from all over the United States received by the National Hansen’s Disease Programs (unpublished observation).

Since *M. lepromatosis* existed in the Americas long before *M. leprae* was introduced, why is it not the dominant pathogen? Considering this question, Lopopolo et al.^4^ suggests that one reason might be observational, since PCR testing for *M. lepromatosis* is not yet done routinely. No large-scale epidemiological studies comparing these two organisms have been conducted, leaving this question open. However, to the best of my knowledge, no published reports to date indicate *M. lepromatosis* is a frequent cause of human leprosy.

Ancestral specimens indicate that millennia ago, *M. lepromatosis* was geographically widely dispersed throughout the Western hemisphere.^5^ One of the ancestral DNA specimens studied was from what is now Canada, also the original home of the patient reported by Olson et al.^1^ Their patient had no identifiable human contact with leprosy but had extensive outdoor exposure, focusing new attention on old theories and controversies about environmental reservoirs or vectors for the mycobacteria that cause leprosy.^6^ Since both of these organisms have existed for millennia, it seems likely that each found significant environmental reservoirs to survive, and appear to have selected different animal hosts. Initial evidence of this was the surprising discovery that armadillos are susceptible to *M. leprae*,^7^ and we now know they share several genotypes of *M. leprae* with humans.^8^ In contrast, *M. lepromatosis* has been found in red squirrels in the United Kingdom^9^, but not in armadillos.

Notably, both of these non-human hosts are mammals. In the early 20^th^ century, all sorts of animals, from eels to pigeons, were inoculated in attempts to develop an experimental model and discover possible animal reservoirs of *M. leprae*.^10^ These efforts all failed. Nevertheless, it seems unlikely that armadillos and red squirrels are the only non-human hosts that have enabled these mycobacteria to survive for so long. Efforts continue to identify possible environmental reservoirs for each of these species.^11^ Additionally, recent studies have revisited the possibility that *M. leprae* and *M. lepromatosis* may survive for some time in soil,^12^ but the infectivity of these isolates is uncertain.

*M. leprae* and *M. lepromatosis* may also differ in their fitness within the human host. Dual infection with both organisms has been observed,^13^ and it is likely that future studies will determine whether one of them predominates in such cases.

Leprosy cannot be eradicated by interruption of human–human transmission alone, at least not in the Western hemisphere. To improve control efforts, a better understanding of environmental reservoirs is essential. After our long history of efforts to understand, treat, and control leprosy, such knowledge may provide future directions for leprosy control. Expect more surprises.
